# Emphysematous pyelonephritis with rare and severe iliac vascular complications: a case report and review

**DOI:** 10.3389/fmed.2024.1512449

**Published:** 2025-01-13

**Authors:** Pan Gao, Han Yang, Zhi Zhang

**Affiliations:** ^1^Department of Urology, Second People's Hospital of Yichang, Second People's Hospital of China Three Gorges University, Yichang, China; ^2^Department of Urology, The Affiliated Hospital of Qingdao University, Qingdao, China

**Keywords:** emphysematous pyelonephritis, diabetes, ureteral calculi, aneurysm, iliac aneurysm

## Abstract

Emphysematous pyelonephritis (EPN) is a rare but severe necrotizing infection often associated with diabetes, ureteral obstruction, and gas production in the renal parenchyma and perinephric area. This report describes a 54-year-old man with type 2 diabetes who presented with right lumbar pain and was diagnosed with EPN complicated by right ureteral calculi and perinephric gas accumulation. Despite initial improvement with fluid resuscitation, antibiotics, and drainage, inadequate blood glucose control led to a worsening of the infection, eventually involving the psoas major muscle and iliac vessels. This progression resulted in the formation of an iliac aneurysm, which required endovascular surgery. The patient’s recovery was supported by proper blood glucose management and targeted antibiotic therapy. This case highlights the importance of early recognition, appropriate management, and strict glycemic control in preventing severe complications of EPN.

## Introduction

Emphysematous pyelonephritis (EPN) is a rare but life-threatening infection of the kidney and perinephric area characterized by the presence of gas in the renal parenchyma, renal pelvis, or perinephric space (1). It is often associated with uncontrolled diabetes, urinary tract obstruction, and immunocompromised states (2). EPN has been linked to high mortality rates if not managed appropriately and in a timely manner (3). In this report, we present a unique case of EPN complicated by severe iliac vascular involvement, which has never been reported in the literature. The pathogenesis, management challenges, and potential mechanisms for the development of such complications are discussed.

## Case presentation

A 54-year-old man with a 10-year history of type 2 diabetes mellitus presented to our hospital with a 5-day history of right lumbar pain. The patient had initially been treated with intravenous anti-inflammatory medication at another hospital, but his symptoms persisted and worsened. On presentation, his vital signs included a temperature of 36.2°C, a heart rate of 107 beats per minute, and a blood pressure of 94/73 mmHg. The patient exhibited obvious percussion pain in the right renal area. Physical examination revealed no other significant findings.

Laboratory tests showed a white blood cell count of 11.3 × 10^9/L, a platelet count of 52 × 10^9/L, and elevated C-reactive protein (CRP) levels of 169.65 mg/L, indicative of systemic inflammation. Renal function tests revealed a serum creatinine level of 179 mmol/L and a blood urea nitrogen level of 18.7 mg/dL, suggesting mild renal impairment. Urinalysis revealed leukocyturia of 2,102/μL and elevated procalcitonin (PCT) of 48.71 ng/mL, indicating severe infection.

**Figure 1:**
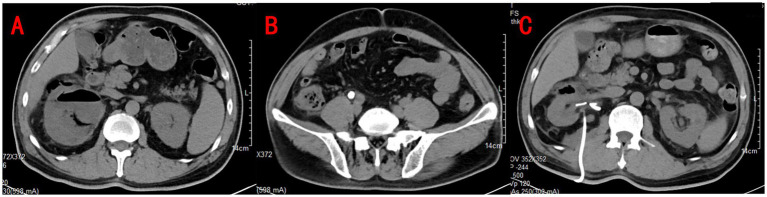
Urinary CT revealed gas in the right renal parenchyma and perirenal with right hydronephrosis **(A)** and right ureteral calculi at the intersection with the iliac vessels **(B)**. Repeat CT showed a significant improvement in right hydronephrosis and pyelonephritis with reduced gas accumulation **(C)**.

A contrast-enhanced CT scan of the abdomen and pelvis revealed right ureteral calculi complicated by emphysematous pyelonephritis (EPN). Gas was seen in the renal parenchyma, renal pelvis, and perinephric space, accompanied by right-sided hydronephrosis (Figures 1A,B). Given the severity of the findings, the patient was immediately started on broad-spectrum intravenous antibiotics and fluid resuscitation. Emergency percutaneous renal puncture drainage was performed, and foul-smelling pus with gas was drained from the right kidney. *E. coli* was cultured from both the urine and the pus, confirming the infectious etiology.

In the following days, the patient’s condition improved with continued antibiotic therapy, aggressive fluid resuscitation, and insulin therapy to control blood glucose levels. After stabilization, the patient underwent a right ureteroscopy, lithotripsy, and double-J stent insertion. A follow-up CT scan performed 1 week later (Figure 1C) showed a significant reduction in hydronephrosis and resolution of perinephric inflammation. At the time of discharge, after 2 weeks of hospitalization, the patient’s laboratory values had normalized, with stable renal function and blood glucose control. No bacterial growth was detected in two subsequent urine cultures. Given the absence of any evidence of infection in the patient, a double-J stent removal procedure was performed 3 weeks after the patient was discharged from the hospital.

**Figure 2:**
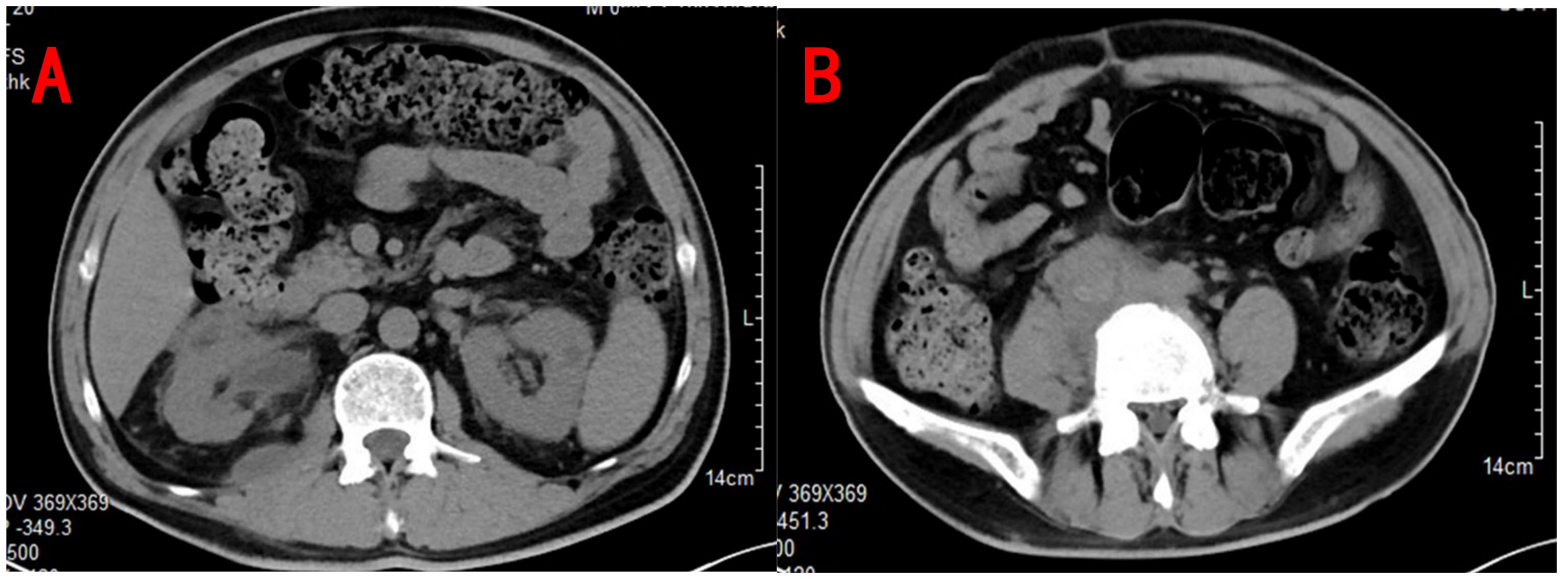
Urinary CT showed slightly more hydronephrosis in the right kidney and more perirenal inflammation than before **(A)**, and soft tissue density shadows are seen around the inferior vena cava and the right common iliac vessels **(B)**.

However, 1 month after discharge, the patient returned to our center with recurrent right lumbar pain. He had not been adhering to the prescribed blood glucose control regimen at home. On physical examination, he had stable vital signs, but his blood glucose level was elevated to 26.9 mmol/L. The laboratory results showed leukocytosis (11.1 × 10^9/L) and leukocyturia (277/μL), suggesting ongoing infection. A new contrast-enhanced CT scan revealed mild progression of hydronephrosis and increased perinephric inflammation (Figure 2). Blood cultures identified multidrug-resistant *E. coli*, prompting a change in antibiotic therapy.

**Figure 3:**
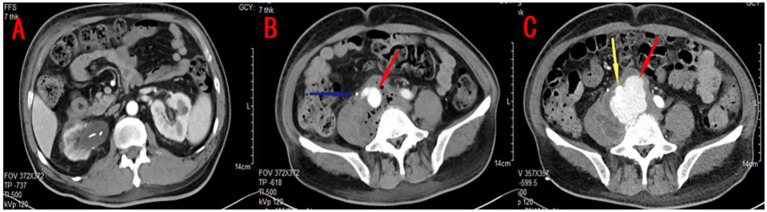
Contrast-enhanced CT findings: **(A)** The boundary between the lesion and the psoas major muscle, as well as the blood vessel, was unclear. A right common iliac aneurysm (red arrow), approximately 2.2 cm in diameter, and a right ureteral stent (blue arrow) were observed. **(B)** The right kidney appeared to be atrophied, irregularly shaped, and surrounded by mild inflammation. **(C)** Follow-up contrast-enhanced CT showed that the enhancement shadow around the iliac artery (yellow arrow) had significantly increased in size compared to the previous scan and exhibited an irregular shape. A mass-like shadow (red arrow) with a density lower than that of arterial blood vessels was observed, suggestive of rupture of the right common iliac aneurysm with localized encapsulation.

Given the recurrence of infection, a double-J stent was reinserted, and the patient was started on insulin therapy for glycemic control. Despite these measures, the patient continued to experience significant right lumbar pain, and his inflammatory markers remained elevated. A contrast-enhanced CT scan performed shortly thereafter revealed a right common iliac artery aneurysm, with gas accumulation and soft tissue shadows adjacent to the right iliopsoas muscle (Figures 3A,B). The inferior vena cava was obscured by the inflammatory mass, suggesting the possibility of a contained rupture, thrombosis, or pneumocystis. The patient and his family hesitated regarding the proposed endovascular treatment of the aneurysm as they were concerned about potential complications.

Surprisingly, 2 weeks later, a repeat contrast-enhanced CT scan (Figure 3C) showed that the aneurysm had ruptured but was contained by a surrounding fibrous capsule, preventing further hemorrhage. Fortunately, the right psoas muscle infection had improved. The patient underwent urgent endovascular surgery to repair the iliac aneurysm. Following surgery, the patient continued with appropriate fluid resuscitation, strict glycemic control, and tailored antibiotic therapy. His condition gradually stabilized, and he was discharged once again after his infection improved.

## Discussion

Emphysematous pyelonephritis (EPN) is a rare but life-threatening infection characterized by the presence of gas in the renal parenchyma, renal pelvis, or perinephric space (4). First described by Kelly and MacCallum in 1962 (1), EPN is most commonly associated with uncontrolled diabetes mellitus, although other risk factors such as urinary tract obstruction, immunocompromised states, and female gender also play a role (5, 6). The pathophysiology of EPN involves the fermentation of glucose by gas-producing bacteria, particularly *Escherichia coli* (*E. coli*), leading to the production of gas and tissue necrosis (7). The disease has a high mortality rate if not promptly diagnosed and managed, especially when complicated by sepsis or other severe infections (8).

In this case, the patient had a 10-year history of type 2 diabetes mellitus, which was poorly controlled, contributing significantly to the development of EPN. The patient’s symptoms—right lumbar pain and fever—were typical of EPN, and the CT scan findings confirmed the diagnosis of right-sided EPN complicated by hydronephrosis and gas accumulation in the renal and perinephric spaces. The presence of gas in the renal parenchyma and perinephric space is a hallmark of EPN, and this finding is associated with a poor prognosis if not addressed promptly (9). Early intervention with broad-spectrum antibiotics, fluid resuscitation, and percutaneous drainage was essential for the patient’s initial recovery (10).

However, the patient’s poor glycemic control after discharge led to the recurrence of the infection and the development of severe complications. Despite initial improvement, the failure to maintain blood glucose within the recommended range resulted in the worsening of the infection, eventually leading to the formation of an iliac aneurysm, a rare but serious complication of EPN. To the best of our knowledge, this is the first reported case of EPN complicated by an iliac aneurysm. The presence of a vascular aneurysm in this setting is particularly concerning as it raises the risk of rupture, which could lead to life-threatening hemorrhage.

The mechanism by which EPN leads to aneurysm formation remains unclear, but it is likely related to the chronic infection and inflammatory process (11). Inflammation of the psoas major muscle and iliac vessels, compounded by high glucose concentrations and tissue ischemia, may have contributed to endothelial damage, leading to the formation of an aneurysm. In addition, the gas-producing bacteria in EPN may further damage the vessel walls, weakening them and facilitating aneurysm formation. In our patient, the iliac aneurysm was initially contained, and the rupture was localized by a fibrous capsule, preventing massive hemorrhage. This contained rupture likely saved the patient’s life and allowed for urgent endovascular repair, which ultimately resulted in a favorable outcome.

In the management of EPN, early diagnosis and intervention are critical to improving patient outcomes. The standard treatment for EPN includes broad-spectrum antibiotics, fluid resuscitation, and effective drainage of the infected kidney (12). In severe cases, such as this one, where there is extensive vascular involvement or other life-threatening complications, multidisciplinary management is essential. Nephrectomy may be necessary in cases of non-functioning or severely damaged kidneys, but it should be reserved for patients who do not respond to medical treatment or when there is concern for ongoing infection or sepsis (13). In our patient, despite the severe complications, conservative management with glycemic control, drainage, and surgery for the aneurysm ultimately resulted in recovery.

This case highlights the importance of strict blood glucose control in patients with diabetes as uncontrolled hyperglycemia is a significant risk factor for the development of EPN. In addition, it underscores the need for the early recognition of complications such as vascular involvement, which may not be apparent at the time of initial presentation. Regular follow-up and imaging are essential to detect recurrence or new complications, as seen in this patient.

The classification of EPN proposed by Huang and Tseng provides useful guidance for the management of the disease (14). Based on their classification system, our patient would be categorized as type IIIb, which involves extensive perinephric and renal involvement with potential complications such as sepsis and vascular involvement. In such cases, a multidisciplinary approach is crucial, with the involvement of nephrologists, urologists, and vascular surgeons to address both the infection and any associated complications.

Finally, this case underscores the importance of patient education and compliance. Given the potential for severe complications, patients with diabetes must be closely monitored and educated about the importance of blood glucose control, adherence to prescribed therapies, and timely medical follow-up.

## Data Availability

The raw data supporting the conclusions of this article will be made available by the authors, without undue reservation.
